# Critical Appraisal of Plain Abdominal X-rays in Acute Abdomen: A Review

**DOI:** 10.7759/cureus.80420

**Published:** 2025-03-11

**Authors:** Rizwan Ahmed, Hadeel Suliman, Beebee Mubarak Jan, Mohit Bhatia

**Affiliations:** 1 General and Colorectal Surgery, Princess Royal University Hospital, King's College Hospital NHS Foundation Trust, London, GBR; 2 General Surgery, King's College Hospital, King's College Hospital NHS Foundation Trust, London, GBR

**Keywords:** abdominal pain, abdominal x-rays, health system development, pain in abdomen, radiation exposure

## Abstract

Abdominal X-rays (AXR) are associated with the surgical specialty, especially for patients presenting to the emergency department. They have been a useful tool for clinicians for the initial assessment of abdominal pain. With the advent of modern radiological modalities, their usefulness has diminished, particularly in developed countries. AXRs are often considered unwarranted in many conditions and are associated with radiation exposure; hence, their use has become limited worldwide, and many studies suggest the careful use of AXRs. AXR should be used more judiciously and not as a routine investigative tool. The benefits and hazards should be considered before performing an X-ray to understand abdominal pain.

## Introduction and background

Abdominal pain is one of the most common presenting features in surgical patients visiting the emergency department. Abdominal X-rays (AXRs) have long been part of the investigation protocol for abdominal pain and are used as an initial screening tool worldwide. However, many recent studies have raised concerns about the excessive use of AXRs and suggest they should be used only in limited conditions, rather than for all patients presenting with abdominal pain [1].

Many authors in the recent past have questioned its effectiveness in concluding a diagnosis. Due to its low diagnostic yield and radiation exposure compared to other newer radiological investigations, its role as a mandatory investigational tool has been questioned. Many guidelines have been published on limiting AXR use in cases of abdominal pain. In 1960, a study concluded that only 7% of AXRs were diagnostic and that these X-rays did not change the clinical diagnosis or influence management [2]. Similar studies have questioned the rationale behind using AXR for all cases of abdominal pain [3]. It is estimated that the use of AXR has gone down by one-third after the wide acceptance and availability of ultrasonography and CT scans [4].

In recent years, the American College of Radiology (ACR) and other concerned societies have advocated for limited use of AXRs and strict adherence to their guidelines, thereby reducing radiation exposure and financial burden on hospitals [5].

Pain in the abdomen is one of the most common presenting complaints in adults attending the emergency department [6]. Abdominal pain can be due to various causes; however, the most common causes worldwide include perforation, renal colic, biliary colic, bowel obstruction, and diverticulitis [7].

Clinical examination, history, and diagnostic tools are important in determining the cause of abdominal pain. AXR is the most common initial radiological investigation offered to patients with acute abdominal pain [8]. However, its irrational use not only leads to increased radiation exposure but also increases the financial burden for both the institution and patients. AXR radiation exposure is greater than that of a chest X-ray [9].

Considering the evidence of the unjustified use of AXR worldwide, the Royal College of Radiology has issued guidelines to safeguard patients' interests. Many studies have implied that these guidelines are not followed and should be strictly adhered to in order to ensure patient safety and reduce economic liability (Table 1) [10].

**Table 1:** Royal College of Radiology guidelines Reference: [10] This table has been presented under the terms of the Creative Commons Attribution License CC-BY 4.0.

Royal College of Radiology iRefer guidelines for the use of plain abdominal radiography
Clinical suspicion of obstruction
Acute exacerbation of inflammatory bowel disease
Palpable mass (specific circumstances)
Constipation (specific circumstances)
Acute and chronic pancreatitis (specific circumstances)
Sharp/poisonous foreign body
Smooth and small foreign body e.g., coin, battery (specific circumstances)
Blunt or stab abdominal injury (specific circumstances)

A study suggested having a designated investigation protocol will enable minimizing radiation exposure and will be cost-effective. It is estimated that around 50% of the X-rays can be avoided by judicious use of clinical acumen and a proper guideline pathway [11].

## Review

Renal and ureteric colic/calculi

Renal and ureteric colic are two of the common causes of visits to emergency departments, which often prompt imaging tests [12]. AXR can help in identifying radio-opaque stones; however, it has limited utility in detecting small stones, ureteric calculi, and radio-lucent stones [13]. It is estimated that average radiation exposure with AXR is 0.5-1 mSv, and ultra-low dose CT (ULD CT) yields more information, especially in ureterolithiasis, as compared to the AXR with comparable radiation exposure [14].

Many studies suggest that AXR is not very specific or sensitive for detecting urolithiasis, and its use as an initial diagnostic tool should be limited. Ultrasound should be the first investigation of choice in young patients, while a CT scan is ideal as it provides more information [15].

A study by McGeorge et al. [16] concluded that the advantages of AXR - being readily available and involving low radiation - are countered by the limited information it provides, its low diagnostic accuracy, and the recommendation that it should not be routinely used in cases of clinical suspicion of ureteric or renal calculi. Some studies have recommended using AXR along with ultrasonography as an adjunct to negate the low diagnostic accuracy of AXR [17].

Some authors concluded that AXR (kidney, ureter, and bladder (KUB) radiography) has a high sensitivity of 87.5% for stones larger than 5 mm, and it is a cost-effective method for monitoring purposes in patients receiving medical therapy for refractory renal/ureteric stones [18].

Hepatobiliary

X-rays are not very useful for diagnosing biliary pathology, such as gallstones or biliary pancreatitis. Only 15% of the gallbladder stones are radio-opaque and are picked up on AXR, thereby confirming its limited utility in gallstone disease [19]. Ultrasonography is the preferred investigation when suspecting cholecystitis and cholelithiasis. Ultrasonography can provide details like wall thickness, stone presence, and fluid collection; hence, it is more informative and can prompt the clinician to act accordingly. Ultrasonography confers the advantage of being radiation-free and can also distinguish gallstones from gallbladder polyps [20].

In pancreatitis, AXR can show findings of colon cut-off sign and sentinel loop sign to support the diagnosis of pancreatitis; however, these findings are not consistent, and therefore the Royal College of Radiology does not support the routine use of AXR in pancreatitis [10].

In 1990, Balthazar [21] introduced the CT severity index for pancreatitis, which could provide more prognostic information regarding the disease process along with information on its associated pancreatic and extra-pancreatic complications. Furthermore, a CT scan is considered more precise in diagnosing pancreatitis. According to the latest Atlanta guidelines, using a CT scan in the early stages of pancreatitis is not very helpful unless exclusion of the diagnosis is required. The ideal time for performing a CT scan is 72 hours after the onset of symptoms [22].

Non-specific abdominal pain

Along with the clinical examination and laboratory tests, imaging modality has also become an integral part of evaluating a patient with acute abdominal pain with conventional AXR performed as the first-line radiological test [23].

Multiple studies have cast doubts regarding the usefulness of AXR in abdominal pain, and their results showed that around 77-78% of patients had negative AXR when done to find the cause of non-specific abdominal pain [24]. A study concluded that only 10.4% of AXRs are conclusive and of diagnostic importance [25].

Moreover, Gerhardt et al. [26] studied the evaluation of non-specific abdominal pain and found the sensitivity and specificity of AXR to be 56% and 81%, respectively.

Intestinal obstruction

AXR is helpful in diagnosing intestinal obstruction. Various studies show the cumulative sensitivity of AXR ranging from 46 to 90.8%, with a specificity of 50%. However, its drawback is that it does not provide additional information about the potential causes and definitive site of obstruction [27].

A large prospective study showed that only 15.8% of AXRs were of diagnostic significance in a large sample size of bowel obstruction and concluded that these X-rays could have been avoided in 42.1% of patients [28]. A study in the NHS United Kingdom revealed cost savings of around 50-60 million in one year when a strict pathway was implemented in requesting AXR [29].

A study by Suri et al. [30] concluded that AXR has a sensitivity and specificity of 77% and 50%, respectively; whereas, a study by Maglinte et al. [31] concluded that AXR has a specificity of 57%. However, some evidence-based studies recommended that AXR should remain the initial investigation in suspected cases of bowel obstruction [32]. According to the ACR, a CT scan is the investigation of choice for intestinal obstruction, as it provides information about the cause and site of obstruction and is more precise in deciding the management plan compared to plain X-ray films [33].

Viscus perforation

The pneumoperitoneum is visualized as free gas under the dome of the diaphragm on plain X-ray abdominal films. However, it is not helpful for detecting air less than 1 mm [34]. Variability exists in estimating the sensitivity of AXR in the pneumoperitoneum. A study by van Randen et al. [35] reported a 15% sensitivity of AXR, while Baker [36] concluded that AXR can detect pneumoperitoneum in 51% of patients. However, CT scans are more useful as they provide the precise location of perforation along with additional information about fluid collection, bowel dilatation, and bowel viability [37].

Field and Morrison [8] suggested that abdominal pain along with evidence of pneumoperitoneum on AXR will usually act as a guide for laparotomy. Another study concluded that the main reason for the high missed rate with AXR in viscus perforation is usually due to technical faults, including poor quality of the film. This study also emphasized that left lateral decubitus radiographs are highly sensitive in picking up pneumoperitoneum [38].

Appendicitis

Rao et al. [39] concluded that 78% of patients with a clinical diagnosis of appendicitis underwent AXR, but no individual gained additional information from plain X-ray films. Appendicitis is a clinical diagnosis, and conventional X-ray films have no diagnostic value; hence, they should be avoided. A Turkish study found that AXR was able to guide only 10% of perforated appendices [40]. This study echoed the importance of clinical judgment in diagnosing appendicitis. We believe AXR should not be routinely used in suspected appendicitis. Contrary to this, Ekere et al. [41] supported the use of AXR in appendicitis.

Studies have shown that if X-ray requests follow guidelines, positive findings occur in about 77% of AXRs [42]. However, AXR should be used judiciously and not as a routine investigation. Anyanwu and Moalypour concluded that proper adherence to guidelines, regular audits, and changes in the training structure can help decrease the overuse or irrational use of AXR in emergency departments [25]. Various authors have implied not using AXR for all cases of abdominal pain, as they are less informative, sources of radiation, and less likely to change treatment plans. Therefore, they should only be used when justified [43]. Imaging modalities such as ultrasonography and CT scans should be used where they can better aid in determining the diagnosis, as they are more informative and support early decision-making and treatment plans [44].

## Conclusions

Judicious use of AXR in cases of abdominal pain should be practiced to minimize unnecessary radiation exposure and be cost-effective. Adhering to local policies and guidelines will also help rationalize the workload on radiology services. Clinicians should be encouraged to use AXR in clinical scenarios where they can provide meaningful information and avoid delays in treatment or repetitive investigations.
